# Proximal Gastrojejunal Reconstruction after Pancreaticoduodenal Resection

**DOI:** 10.1155/2012/976268

**Published:** 2012-02-22

**Authors:** M. Wayne, A. Cooperman, R. Narang, B. Abbadessa, J. Bratcher, W. Brown, J. Steele, F. Kasmin

**Affiliations:** The Pancreas Center, Beth Israel Medical Center, 37 Union Square West, New York, NY 10003, USA

## Abstract

*Introduction*. Reconstruction by proximal gastrojejunostomy, and distal biliary and pancreatic anastomoses is infrequently used after resection of the head of the pancreas because of fear of fistulas and cholangitis. Pancreaticoduodenectomy is being performed more frequently for cystic malignant and premalignant lesions. Because of this there is a need for endoscopic visualization and biopsy of the residual pancreatic duct, since multi-centricity is characteristic of some of these malignancies. Since endoscopic access of the bile duct and pancreatic duct is difficult and unsuccessful in 50–70% after B II or Roux Y reconstruction, we prospectively studied the merit and complications (early and late) of proximal gastrojejunal (PGJ) reconstruction after pancreaticoduodenal resection. *Material and Methods*. Thirty nine consecutive, non-radomized patients underwent pancreaticoduodenectomy and PGJ reconstruction over 14 mos. There were 21 males and 18 females. *Results*. 7 patients with IPMN have undergone repeat CT scanning for surveillance, with 3 requiring repeat EUS and ERCP. There were no technical difficulties accessing the pancreas or the pancreatic duct, supporting the PGJ reconstruction. *Conclusion*. Proximal gastrojejunal reconstruction following pancreaticoduodenal resection may be safely done with similar morbidity to traditional pancreaticojejunal reconstructions. PGJ reconstruction may be of greater value when direct visual access to the bile duct or pancreatic duct is necessary, and should be considered when doing resection for mucinous cysts or IPMN of the head of the pancreas.

## 1. Introduction

Alimentary reconstruction during pancreaticoduodenal resection invariably places the gastric or duodenal jejunal anastomoses distal to the biliary and pancreatic anastomoses [1, 2]. Despite absence of supporting literature this is done for alleged fears of pancreatic and biliary fistulas that will delay oral feedings and prolong hospitalization. With more PDRs being done for premalignant and malignant pancreatic cystic and ductal lesions, surveillance of the remnant pancreatic duct mucosa for multicentricity may be necessary [3]. We undertook this study to determine the value and risk of proximal B1 gastrojejunal reconstruction.

 There has been an increase in cystic malignant and premalignant lesions being treated by pancreaticoduodenal resection. Because of this there is a need for endoscopic visualization and biopsy of the residual pancreatic duct for future surveillance because multicentricity is characteristic of some of these malignancies. Endoscopic access of the bile duct and pancreatic duct is difficult and unsuccessful in up to 50–70% of cases after Billroth II (B2) or Roux-en-Y reconstruction. We prospectively studied the merit and complications of proximal gastrojejunal (PGJ) reconstruction after pancreaticoduodenal resection. We believe this facilitates endoscopic access to the pancreas and pancreatic duct, which is necessary during surveillance.

## 2. Material and Methods

Thirty-nine consecutive, nonrandomized patients underwent pancreaticoduodenectomy and PGJ reconstruction over 14 months. There were 21 males and 18 females. The mean age was 61.8 ± 12.6 years. Preoperative CT scans, ERCP, and endoscopic ultrasound (EUS) were performed to establish resectability. The indications for surgery were: carcinoma of the pancreas in 26 patients five of whom (12.5%) were initially unresectable and downstaged with neoadjuvant chemotherapy. Seven other patients had main duct intraductal papillary mucinous neoplasms (IPMN); 2 had ampullary cancers, one a gastrointestinal stromal tumor (GIST) tumor involving the pancreas and duodenum, and 3 had distal common bile duct cancers. After standard pancreaticoduodenal resection the jejunal limb is measured and the anastomoses are performed. The biliary anastomosis is performed first in a duct-to-mucosa fashion. This is followed by the pancreatic anastomosis, which is constructed in a duct-to-mucosa fashion and a second layer suturing serosa of jejunum to capsule of pancreas. The final anastomosis is the gastrojejunal, which was made with a GIA stapling device, after first resecting a portion of the stomach. All the anastomoses are retrocolic. Postoperatively, on endoscopic examination, the order is gastric, pancreatic, and then biliary, all accessible via a single efferent limb from the stomach. The anastomoses are approximately 10 cm apart from each other.

## 3. Results

There was no operative peri-operative mortality. The mean operative time was 178 min ± 21 min, and estimated blood loss 262 cc ± 116 cc. There were no intraoperative transfusions given. The postoperative morbidity was (48%) and was of surgical significance in 3 patients (7%). The three included a visceral artery pseudoaneurysm in 1 patient (2.5%), an embolic event to an intestinal branch of the superior mesenteric artery in 1 patient (2.5%) and a gastrojejunal leak in 1 patient (2.5%). The latter two complications required reoperation. Other complications included a type A pancreatic fistula in 2 patients (5%), delayed gastric emptying in 1 patient (2.5%), pneumonia in 3 patients (7.5%), superficial wound infection in 4 patients (10%), atelectasis in 3 patients (7.5%) and *Clostridium difficile* colitis in 2 patients (5%). Readmission after discharge was necessary in 1 patient. One patient with adenocarcinoma of the head of the pancreas developed recurrence with biliary and gastric obstruction 11 months after surgery and required a reoperation. One patient required endoscopic dilatation of a symptomatic pancreaticojejunal anastomotic stricture 10 months after surgery.

All 7 patients with IPMN have undergone repeat CT scanning for surveillance, with 3 requiring repeat EUS and ERCP. There were no technical difficulties accessing the pancreas or the pancreatic duct, supporting the PGJ reconstruction.

## 4. Discussion

Despite a century of surgery for periampullary cancer, survival remains dismal despite significant reduction in operative mortality and major morbidity [4, 5]. Operative preferences and techniques for PDR are many and for the most part do not impact on long-term survival [2] The ultimate determinant of outcome is systemic metastases present long before tumor discovery [2, 6]. Reconstruction after resection is invariably done by proximal biliary and pancreatic enteric anastomoses and distal gastric or duodenal enteric anastomoses. Proximal placement of the gastro or duodenal jejunal anastomosis is neither advocated nor reported because of unsubstantiated concern that a pancreatic or bile leak distal to the stomach or duodenum would cause sepsis or cholangitis, delay oral feeding, and prolong hospitalization. This has been a long-standing, oral, preceptor-based surgical tradition. An extensive literature search of published case reports and case series as well as retrospective and prospective studies over the past 10 years did not result in any citations referencing proximal placement of the gastroenteric anastomosis during PDR. There has been little reason or need to question this since ERCP after PDR for pancreatic cancer is infrequently needed. Pancreatic fistulas are not uncommon after PDR and range from 6% to 25%, most of which close spontaneously, without sepsis or therapy (type A) [7]. The two pancreatic fistulas in this series closed spontaneously and the B1 arrangement did not delay or further complicate this. A small, unreported previous personal experience with pyloric preservation and proximal duodenal jejunal anastomoses was stopped because of delayed gastric emptying. The proximal placement of the duodenojejunostomy in that experience was noncontributory. With the increase in pancreatic incidental lesions found by body imaging, a number of pancreatic resections are being done for intraductal papillary mucinous neoplasm (IPMN), a lesion that creates concerns because of its multicentricity and clinical recurrence after segmental pancreatic resection [8, 9].

The 3 surgical complications in this report warrant comment. One, an embolic episode occurred after dissecting the superior mesenteric artery, necessitating a small bowel resection, and resulting in a complicated and lengthy course. This was unrelated to the gastric reconstruction. A direct complication, a gastric leak from a stapled corner of the transected stomach, prolonged hospitalization and required operative closure. This is infrequent in our experience and is independent of the type of gastric anastomosis. Local recurrence of pancreatic cancer causing obstruction of the biliary or gastric anastomoses and requiring surgery is not frequent. At least 30% of PDR done for pancreatic cancer will recur locally in the bed of resection [2, 10]. When intervention is needed for biliary obstruction, the percutaneous approach is preferred. In this instance, 1 of 26 patients (4%) that underwent B1 gastrojejunostomy required surgery after attempts at endoscopic therapy failed to stent the obstruction.

In 4/7 IPMN patients, postoperative surveillance raised a concern about the remnant pancreatic duct. In each instance ERCP cannulation was successful and the duct was visualized directly in three patients. Endoscopic cannulation of the biliary and pancreatic duct after a Roux en Y reconstruction or a B2 with an afferent limb of varying length is more difficult and unsuccessful in >50% of attempts (kinking, angulations, and scarring of the afferent limb mar success [11, 12]). Success rates as low as 8% have been reported post Whipple ERCP and the difficulties complications are well stated in a website summarizing the difficulties of ERCP with native and altered PD and BD anatomy after B2 resection [13, 14]. Chahal et al. noted a success rate of 51% in 45/88 ERCP attempts after pancreaticoduodenectomy. Success was much more likely for biliary indications, 37/44 patients (84%), than for pancreatic indications, 3/37 patients (8%) [13].

In view of the fact that local recurrence after resection for cancer of the pancreas is at least 30% and there is a chance that this can cause gastric outlet obstruction that may be difficult to treat endoscopically we will use B1 reconstruction selectively, particularly for main duct IPMN.

## 5. Conclusion

Proximal gastrojejunal reconstruction following pancreaticoduodenal resection may be safely done with similar morbidity to traditional pancreaticojejunal reconstructions. PGJ reconstruction may be of greater value when direct visual access to the bile duct or pancreatic duct is necessary, and should be considered when doing resection for mucinous cysts or IPMN of the head of the pancreas. Concerns about pancreatic fistula delaying feeding were not evident in this study. Previous experience with this reconstruction and type A or B pancreatic fistula has shown this to be a theoretical concern. To date it has been a value in 4 of 7 patients.
